# Fault Diagnosis of Medium Voltage Circuit Breakers Based on Vibration Signal Envelope Analysis

**DOI:** 10.3390/s23198331

**Published:** 2023-10-09

**Authors:** Yongbin Wu, Jianzhong Zhang, Zhengxi Yuan, Hao Chen

**Affiliations:** 1 School of Electrical Engineering, Southeast University, Nanjing 210096, China; wuyongbin@seu.edu.cn (Y.W.); 220213013@seu.edu.cn (Z.Y.); 2 Nanjing Power Supply Branch Company, State Grid Jiangsu Electric Power Co., Ltd., Nanjing 211102, China; pingfengma@126.com

**Keywords:** medium voltage circuit breaker, fault diagnosis, vibration signal, envelope analysis, wavelet packet decomposition

## Abstract

In modern power systems or new energy power stations, the medium voltage circuit breakers (MVCBs) are becoming more crucial and the operation reliability of the MVCBs could be greatly improved by online monitoring technology. The purpose of this research is to put forward a fault diagnosis approach based on vibration signal envelope analysis, including offline fault feature training and online fault diagnosis. During offline fault feature training, the envelope of the vibration signal is extracted from the historic operation data of the MVCB, and then the typical fault feature vector *M* is built by using the wavelet packet-energy spectrum. In the online fault diagnosis process, the fault feature vector *T* is built based on the extracted envelope of the real-time vibration signal, and the MVCB states are assessed by using the distance between the feature vectors *T* and *M*. The proposed method only needs to handle the envelope of the vibration signal, which dramatically reduces the signal bandwidth, and then the cost of the processing hardware and software could be cut down.

## 1. Introduction

Medium voltage circuit breakers (MVCB) play a vital role in modern power systems or new energy power stations, and its operating state will affect the stability and power supply reliability of the power grid [1,2]. Numerous studies have shown that mechanical faults are one of the main problems influencing the operational reliability of the circuit breaker (CB) [3,4,5]. In order to monitor the status and realize a fault diagnosis of the CB, the commonly used acquisition signals may include transient electric field [6], vibration signal [7,8,9,10], current signal [9,10,11], sound signal [12], and travel-time curve [13,14]. Among them, the use of vibration signals for CB fault diagnosis has the advantages of low cost and high diagnosis performance. However, in order to reliably switch on or switch off the circuit, it is important to timely and correctly discriminate among the operating states of the CB and find hidden dangers by analysing the mechanical vibration signal produced during the HVCB operation.

The vibration of the CB is a time-varying, non-steady and non-linear signal. A complex vibration signal is divided by empirical mode decomposition (EMD) into several intrinsic mode functions that each contain local characteristic timescales. The improved EMD energy entropy was selected in [15] to obtain the fault feature of the CB, and a genetic algorithm-based support vector machine (SVM) was applied to classify the CB states. However, the traditional EMD has the disadvantages of border effects, model overlap, and the deficiency of strict theory deduction. In order to make the signal continuous over various time scales, ensemble EMD adds Gaussian white noise to the vibration signal, which can solve the problem of EMD mode aliasing. In [16,17], the EEMD and Hilbert marginal spectrum energy entropy were proposed to extract feature vectors, and the SVM was utilized to classify the mechanical faults of the CB. Nevertheless, the ensemble EMD of the vibration signal still leaves certain noise components, and they will influence the fault diagnosis result. The complete ensemble EMD with adaptive noise could eliminate the model aliasing of the ensemble EMD. In [18,19], the complete ensemble EMD with adaptive noise decomposed and reconstructed the intrinsic mode function to obtain a time–frequency matrix. The variational model decomposition was applied in [20,21,22,23], to extract fault features and address the multi-scale entropy of the reconstructed signal, where variational model decomposition can split the vibration signal up into a range of bandwidth-constrained modal functions. However, the mode number is too arbitrary and lacks a general theoretical foundation in decomposition and reconstruction. The wavelet packet decomposition (WPD) has the advantage of high resolution in the whole frequency band, and feature decomposition of the vibration signals is realized in different time periods and frequency bands. For the WPD algorithm, Ref. [24] used the wavelet packet sample entropy and energy spectrum to obtain the feature vectors of vibration signals. Ref. [25] selected the energy rate in time–frequency Eigen-space of wavelet packets as the basis for the fault diagnosis of the CB. In papers [15,16,17,18,19,20,21,22,23,24,25], the existing fault diagnosis methods need to construct fault features based on the complete operating vibration signal, where the signal processing algorithm is complex, the diagnosis speed is slow, and the hardware and software costs are high.

This paper put forwards a fault diagnosis approach for the MVCB based on vibration signal envelope analysis. The proposed approach has the advantages of accelerating recognition speed, decreasing the expenditure on the hardware and software. The major contributions of this research can be concluded as below:

(1) Only the envelope of the vibration signal needs to be processed. The bandwidth of the processed signal for the proposed method is greatly decreased, which reduces the expenditure on software and hardware.
(2) The performance efficiency of the proposed approach is much more than the existing typical fault diagnosis methods, which is adequate for the online fault diagnosis of the MVCB.

The rest of this paper is formed as below. Section 2 discusses the experiment setup and data acquisition system. The fault diagnosis method is introduced in Section 3. The experiment results and discussions are shown in Section 4. Finally, the conclusions of this article are summarized in Section 5.

## 2. Experiment Setup and Data Acquisition

**Figure 1:**
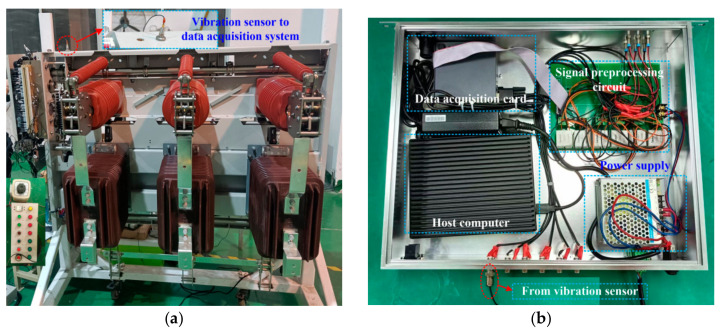
Experiment setup of MVCB. (**a**) MVCB; (**b**) Data acquisition system.

Figure 1 shows the experiment setup which is used to collect the vibration signal at various operating states.

In this paper, a solid-insulated indoor 40.5 kV 630A vacuum MVCB with spring actuator made by Chinese company (Yunfeng Science & Technology Co. Ltd., Jiangyin, China) is adopted, as shown in Figure 1a. A vibration sensor is installed on the crossbeam at the upper side of the MVCB, and it is closed to the operating mechanism of the MVCB. Figure 1b shows the data acquisition system, which includes power supply, signal preprocessing circuit, data acquisition card and host computer. The signal preprocessing circuit could collect and convert the current signal of the vibration sensor into the voltage signal.

The configuration of the data acquisition system is given in Table 1, where the signal sampling frequency is selected as 125 kHz and the data acquisition card is connected to the host computer with USB 2.0.

The MVCB performed switch-on and switch-off operations at six typical cases, including normal case, iron core jamming, shaft-bearing screw looseness, closing-spring relaxation, low relay coil voltage, and relay high-resistance cases. The normal case and five faulty cases (iron core jamming, bearing-screw looseness, closing-spring relaxation, low relay coil voltage, and relay high resistance) studied in this paper are defined as Case 1, Case 2, Case 3, Case 4, Case 5, and Case 6, respectively, as shown in Table 2. In this paper, five faulty cases are emulated in the lab by suspending a heavy weight on the iron core of the closing relay, loosening one bearing screw of the main drive shaft, loosening spring-end nuts, reducing control voltage, and adding a resistor in the relay coil, respectively. The fault reason and emulation method for five faulty cases are also given in Table 2.

**Figure 2:**
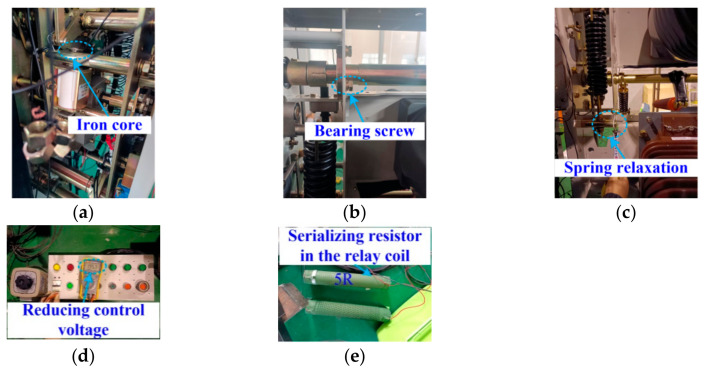
Fault emulation methods. (**a**) Iron core jamming; (**b**) Bearing-screw looseness; (**c**) Closing-spring relaxation; (**d**) Low relay coil voltage; (**e**) Relay high resistance.

Figure 2a shows the emulation method for the iron core jamming case, where a 54 g weight is hung on the moving iron core by the lead, turning the drag force around. Figure 2b shows the emulation method for the bearing-screw looseness case, where one screw of the drive shaft is released by one turn. As the fault reason of the closing-spring relaxation case is due to the aging of the closing spring, the closing-spring relaxation state could be emulated by adjusting the end nuts to decrease the pretension force of the closing spring, as shown in Figure 2c. Figure 2d shows the emulation method for the low relay coil voltage case, where the control voltage is decreased to 0.9 times the rated voltage. As the fault reason of the relay high-resistance case is due to the relay coil aging or the poor contact of relay, the relay high-resistance case could be emulated by adding a 5 ohm resistor into the circuit of the closing relay coil, as shown in Figure 2e.

For mechanical circuit breakers, the closing spring stores energy for the opening spring during the closing operation. The vibration signal energy of the closing process is greater than that of the opening process, and the fault features of the vibration signal during the closing process are more significant than those during the opening process. Therefore, the vibration signal of the MVCB during the closing operation is collected and studied in this paper. Fifteen sets of vibration signal per case are measured by a data acquisition system. As shown in Table 3, 10 sets of samples (named Database 1) are randomly selected to offline train fault feature, and the remaining five sets of samples (named Database 2) were used for testing to verify online fault diagnosis algorithm.

**Figure 3:**
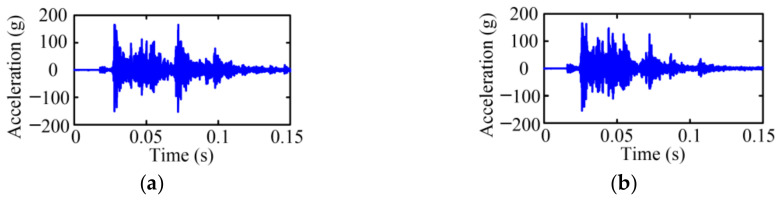
Time-domain vibration signal of the MVCB. (**a**) Normal case; (**b**) Closing-spring relaxation case.

Figure 3 gives the vibration signals of the MVCB in the time domain under the closing operation, where the vibration signals present strongly non-linear and non-steady properties. The operation process of the MVCB is transitory, and the duration is generally about several tens of millisecond. As shown in Figure 3, it is obvious that two processes are carried out under different states but it is difficult to discriminate the state only by the time-domain signals.

**Figure 4:**
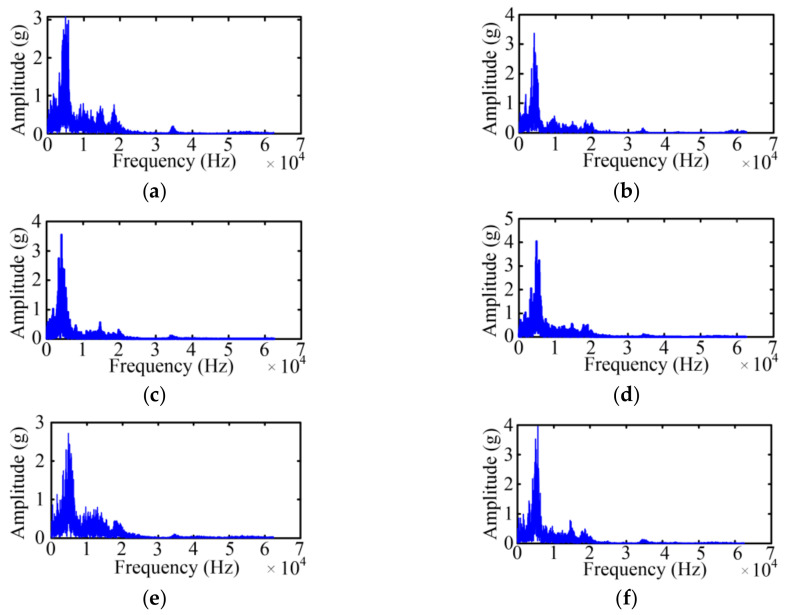
Spectrum diagrams of the vibration signal. (**a**) Case 1; (**b**) Case 2; (**c**) Case 3; (**d**) Case 4; (**e**) Case 5; (**f**) Case 6.

According to the Fast Fourier Transform (FFT) of the original vibration signal, Figure 4 shows the spectrum diagrams of the original vibration signal under six states, where frequency band width is quite large and the main spectrum is within 0–20 kHz. Compared with the five faulty states, the spectrum of Case 1 is richer and has higher wave crests.

Meanwhile, if researchers can select the appropriate feature vector in the frequency spectrum diagram, it will be relatively easy to distinguish the four states by the frequency domain signals. In [7,8], the researcher usually selected four representative vibration feature as the frequency domain features, and the specific feature variables and expressions are shown in Table 4. Here, *L* is the length of one set sample at 125 kHz. *S*(*f*) is the amplitude of the frequency *f* in the frequency spectrum. *C_avf_* is the average value of the frequency spectrum. *C_rmsf_* is the root mean square value of the frequency spectrum. *C_sdf_* is the standard deviation value of the frequency spectrum. *C_gcf_* is the center of gravity value of the frequency spectrum.

In [13,18,19], the extreme learning machine (ELM) is applicable to the small sample classification problem in fault diagnosis of the MVCB. The ELM is used to distinguish the six cases of the MVCB in accordance with the selected frequency domain features. Database 1 is adapted to train the ELM model, and Database 2 is used to test the ELM model. The details of the MVCB state identification are shown in Table 5. The average training accuracy is 91.67%, and the average testing accuracy is 76.67% by using the selected features of frequency domain and ELM.

## 3. Envelope Analysis of Vibration Signal

**Figure 5:**
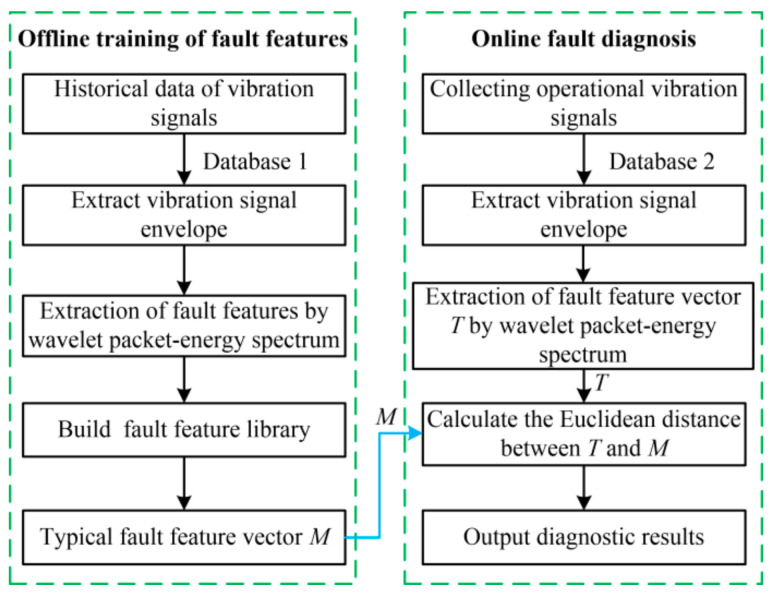
Fault diagnosis structure based on vibration signal envelope analysis.

From the frequency domain signal, four states of MVCB could be discriminated, but the complete operating vibration signal must be processed. To accelerate diagnosis speed and decrease the expenditure on hardware and software, a fault diagnosis approach based on vibration signal envelope analysis is proposed, as shown in Figure 5. The fault diagnosis process consists of offline training of fault features and online fault diagnosis.

In the offline training part, the vibration signal of MVCB during closing under four states is collected by the data acquisition system. The Hilbert transform (HT) is used to extract the envelope of the vibration signal. The wavelet packet energy spectrum is adopted to acquire the fault feature of the vibration signal envelope. The fault feature library is built by the fault feature and fault label of the MVCB. The typical fault feature vector *M* is calculated by the fault feature library.

The online fault diagnosis part includes collecting the operating vibration signal, extracting the vibration signal envelope, and acquiring the fault feature vector *T* of the vibration signal envelope based on the wavelet packet energy spectrum. Then, the selected fault feature *T* and the typical fault feature vector *M* are input to the distance discrimination block to calculate the fault condition, which shows the case of the MVCB.

### 3.1. Vibration Signal Envelope

The continuous time signal is assumed to be *x*(*t*) for the sake of describing the HT, and the HT of *x*(*t*) can be expressed as

(1) $$y(t)=\frac{1}{\pi }\int_{-\infty }^{+\infty }\frac{x(\tau )}{t-\tau }d\tau =\frac{x(t)}{\pi t}$$

where *y*(*t*) is the HT of *x*(*t*).

Using *x*(*t*) and *y*(*t*), a new analytical signal *h*(*t*) can be constructed as

(2) h(t)=x(t)+jy(t)

The magnitude *a*(*t*) of *h*(*t*) is described as

(3) $$a(t)=\left|h(t)\right|=\sqrt{x^{2}(t)+y^{2}(t)}$$

where *a*(*t*) is the envelope of *x*(*t*).

### 3.2. Fault Feature Extraction

**Figure 6:**
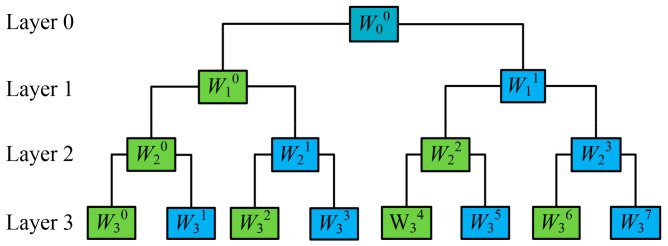
Schematic diagram of the WPD.

The vibration signal of the MVCB is composed of multiple instantaneous non-steady vibration events. WPD is a practical way to process non-linear and non-steady signals [15]. Compared with the wavelet analysis, WPD has a higher time–frequency resolution and stronger localization performance in all frequency bands. Figure 6 shows the structure of a three-layer WPD, in which the *n*-th level and *l*-th sub-band of the reconstructed wavelet coefficients *W_n_^l^* represent 1/2*^n^* of the frequency information.

After the vibration signal envelope is decomposed by the WPD, the energy value of each frequency band is calculated as

(4) $$E_{j}^{*}=\sum_{k=1}^{N}W_{n}^{l}(k{)}^{2}\;\;\;\;\;l=0,1,\cdots ,2^{n}-1;\;j=1,2,\cdots ,2^{n}$$

where *n* is the amount of WPD layers, and *N* is the amount of WPD coefficients in *W_n_^l^*.

The energy value of each frequency band for the *n*-layer WPD of the vibration signal envelope is:

(5) $$E^{*}=(E_{1}^{*},E_{2}^{*},\cdots ,E_{j}^{*},\cdots ,E_{2^{n}}^{*})$$

The energy value vectors are normalized to gain the fault feature of the MVCB, which is in the form of

(6) $$\begin{array}{l} E=(E_{1}^{*}/\sum ,E_{2}^{*}/\sum ,\cdots ,E_{j}^{*}/\sum ,\cdots ,E_{2^{n}}^{*}/\sum )\\ \;=(E_{1},E_{2},\cdots ,E_{j},\cdots ,E_{2^{n}}) \end{array}$$

where $\sum =E_{1}^{*}+E_{1}^{*}+\cdots +E_{j}^{*}+\cdots +E_{2^{n}}^{*}$.

For the offline training part, the fault feature library is composed of the fault feature and the label of four states of the MVCB. The author takes out *g* groups of feature vectors in the same case, adds them to each frequency band and averages them as typical feature vectors. Then, the fault feature vector of a typical case can be expressed as

(7) $$\begin{array}{l} M_{i}=(m_{1},m_{2},\cdots ,m_{j},\cdots ,m_{2^{n}})\\ \;=\frac{1}{g}(\sum_{i=1}^{g}E_{1}(i),\sum_{i=1}^{g}E_{2}(i),\cdots ,\sum_{i=1}^{g}E_{j}(i),\cdots ,\sum_{i=1}^{g}E_{2^{n}}(i)) \end{array}$$

The typical fault feature vector *M* is defined as

(8) $$M={\left[M_{1};M_{2};\cdots ;M_{i};\cdots ;M_{p}\right]}^{T}$$

where *p* is the amount of typical cases.

For the online fault diagnosis process, the fault feature vector *T* can be described as

(9) $$T=(t_{1},t_{2},\cdots ,t_{j},\cdots ,t_{2^{n}})=(E_{1},E_{2},\cdots ,E_{j},\cdots ,E_{2^{n}})$$

### 3.3. Fault Discrimination

The distance discrimination is to establish the distance *d*(*T*, *M_i_*) from the judgment object *T* to *M_i_*. The distance *d*(*T*, *W_i_*) adopts the Euclidean distance, and the expression of the Euclidean distance is

(10) $$d(T,M_{i})=\sqrt{\sum_{q=1}^{2^{n}}{\left(t_{q}-m_{q}\right)}^{2}}$$

The discriminant function is defined as

(11) $$W(r,T)=d(T,M_{i})$$

The fault state is discriminated according to the principle of the closest distance. There is:

(12) $$W(z,T)=\min\{W(r,T)|r=1,2,\cdots ,p\},T\in M_{z}$$

## 4. Results and Discussions

**Figure 7:**
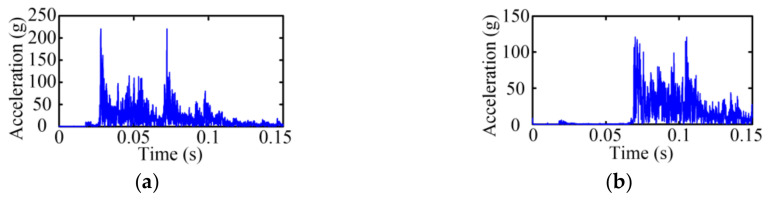

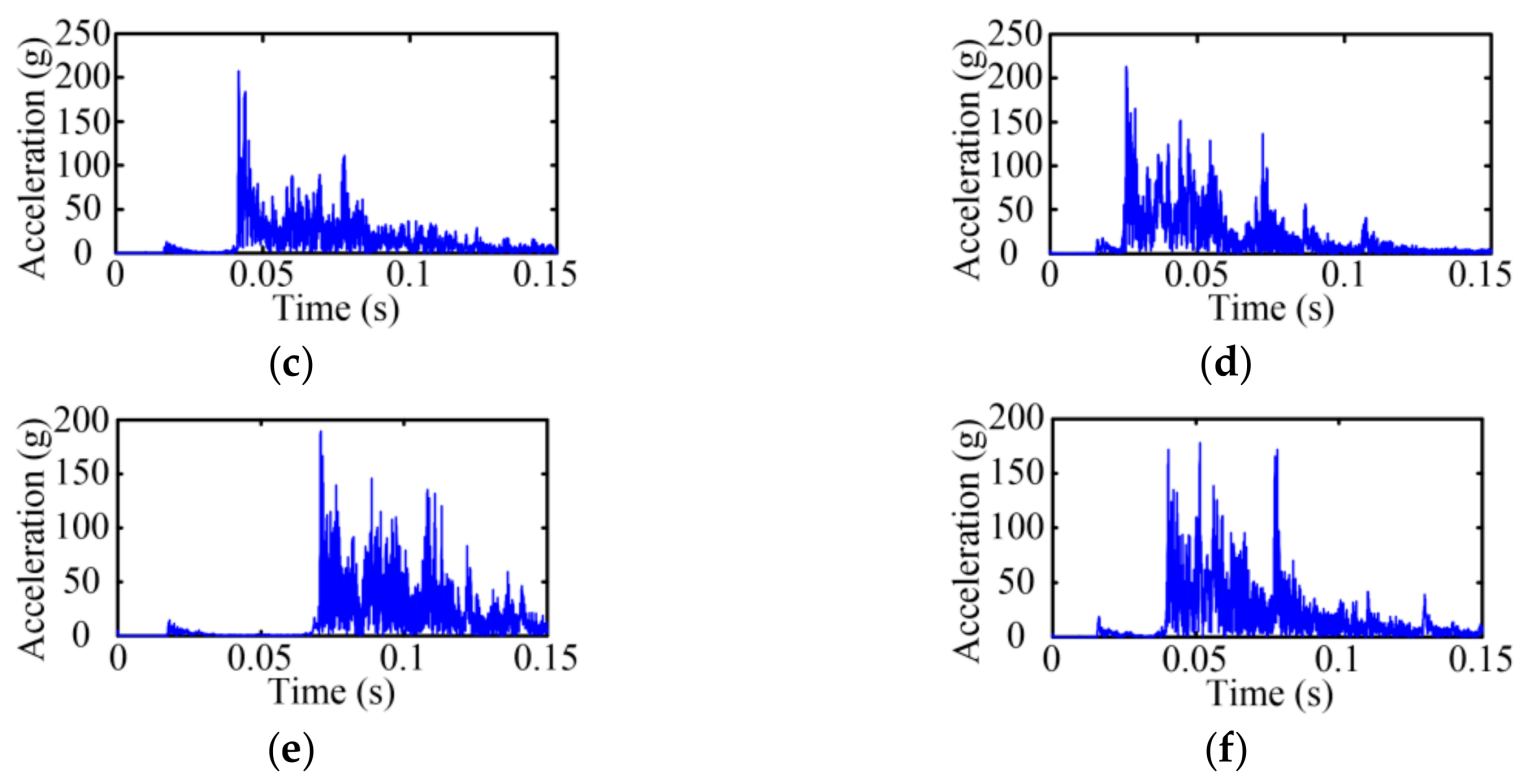
Vibration signal envelope under four cases. (**a**) Case 1; (**b**) Case 2; (**c**) Case 3; (**d**) Case 4; (**e**) Case 5; (**f**) Case 6.

To evaluate whether the suggested fault diagnosis approach is effective, Database 1 is selected for offline training of fault features, while Database 2 is used for online fault diagnosis. The vibration signal envelope is extracted by the HT method under six cases, as shown in Figure 7, where the vibration signal envelopes under six cases are different.

**Figure 8:**
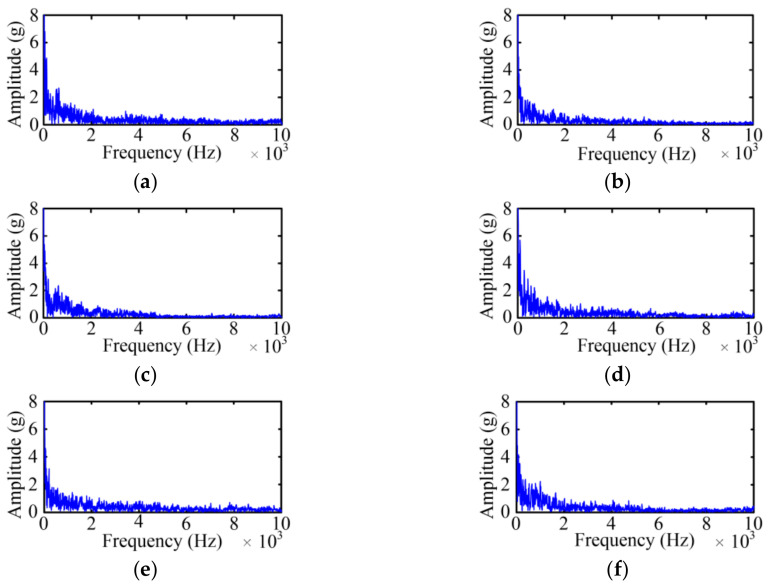
Vibration signal envelope spectrum under four cases. (**a**) Case 1; (**b**) Case 2; (**c**) Case 3; (**d**) Case 4; (**e**) Case 5; (**f**) Case 6.

Based on the vibration signal envelope, the FFT is adopted to acquire the spectrum of the vibration signal envelope. As shown in Figure 8, the frequency band width of the vibration signal envelope is quite small and the main frequency spectrum is within 0–4 kHz, which is less than the frequency spectrum of the original vibration signal. In other word, the bandwidth of the processed signal is greatly decreased.

**Figure 9:**
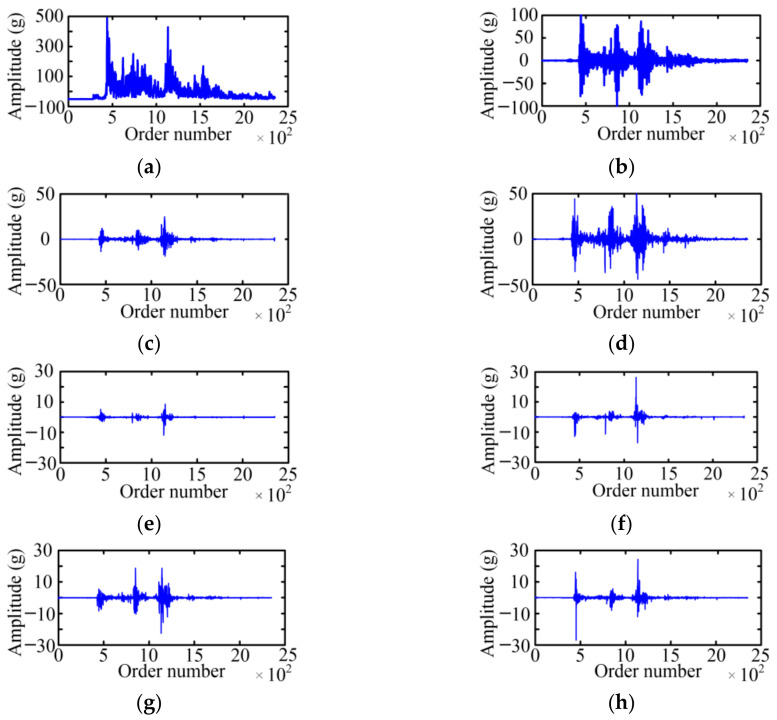
The WPD result of the normal case. (**a**) *W*_3_^0^; (**b**) *W*_3_^1^; (**c**) *W*_3_^2^; (**d**) *W*_3_^3^; (**e**) *W*_3_^4^; (**f**) *W*_3_^5^; (**g**) *W*_3_^6^; (**h**) *W*_3_^7^.

The WPD is used to acquire the fault feature on the basis of a vibration signal envelope. The selected wavelet basis function and decomposition layer will affect the decomposition result of the WPD. For the wavelet basis function, the Symlet 5 is selected as the wavelet basis function of the WPD with the large tests. For the decomposition layer, when the number of selected decomposition layers is small, the analysis speed is fast, which is especially obvious for high-frequency signals, but the analysis precision is low. Considering the relationship between the decomposition layer and analysis effect, the paper adopts a three-layer WPD for six typical cases of MVCB. Figure 9 shows the WPD result of the normal case of the MVCB. Eight WPD coefficients correspond to eight frequency bands, and the amplitude of the WPD coefficient in the first four frequency bands is much greater than the latter four frequency bands.

**Figure 10:**
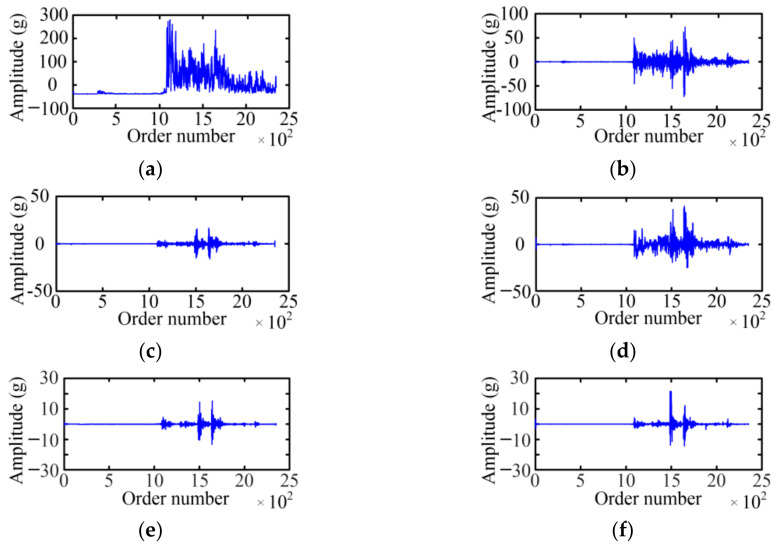

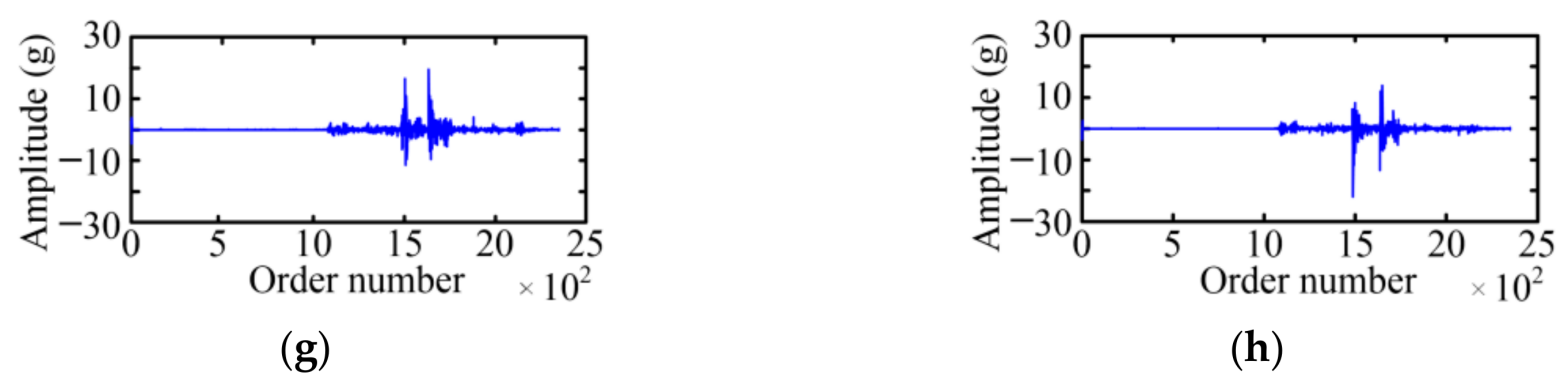
The WPD result of the iron core jamming case. (**a**) *W*_3_^0^; (**b**) *W*_3_^1^; (**c**) *W*_3_^2^; (**d**) *W*_3_^3^; (**e**) *W*_3_^4^; (**f**) *W*_3_^5^; (**g**) *W*_3_^6^; (**h**) *W*_3_^7^.

**Figure 11:**
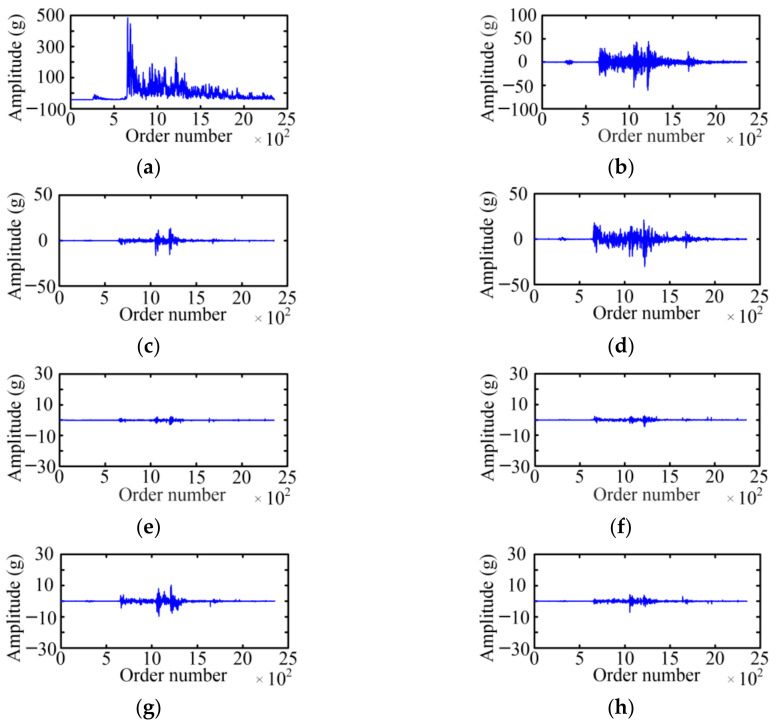
The WPD result of the bearing-screw looseness case. (**a**) *W*_3_^0^; (**b**) *W*_3_^1^; (**c**) *W*_3_^2^; (**d**) *W*_3_^3^; (**e**) *W*_3_^4^; (**f**) *W*_3_^5^; (**g**) *W*_3_^6^; (**h**) *W*_3_^7^.

**Figure 12:**
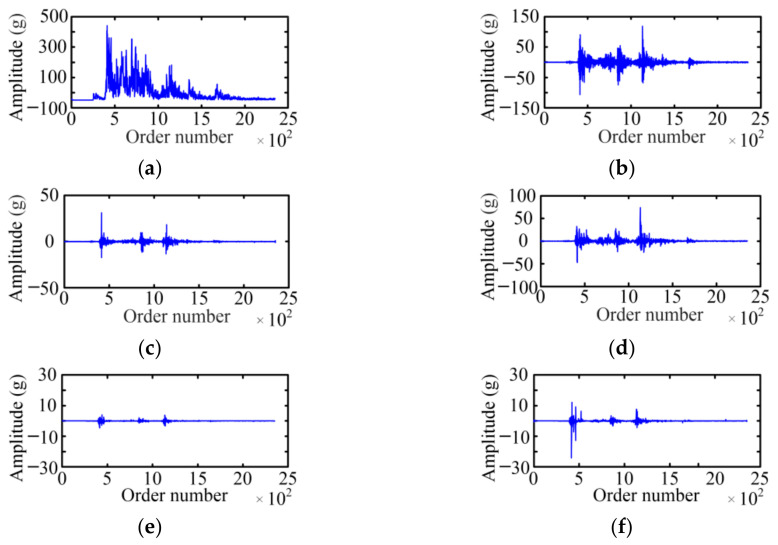

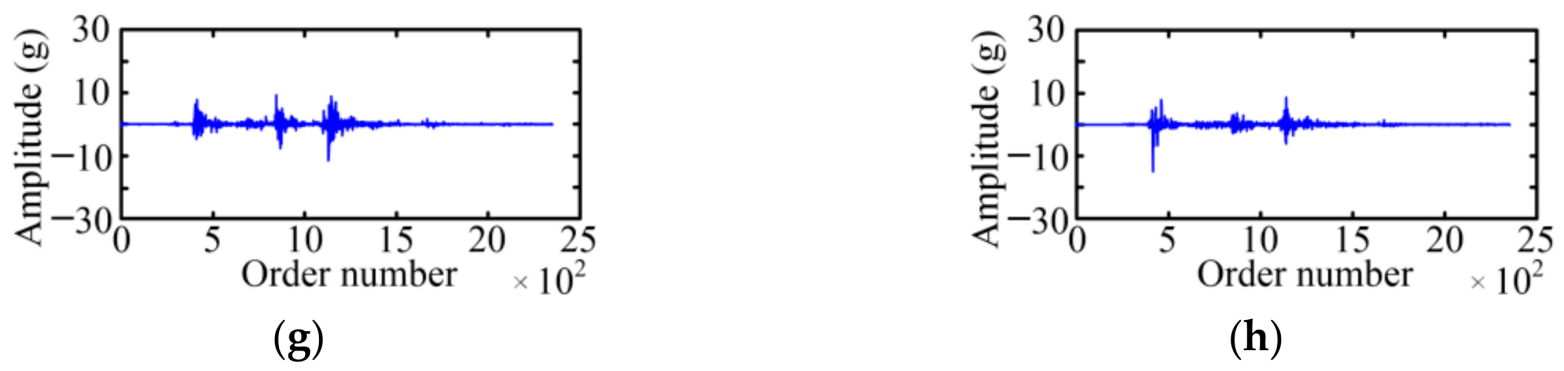
The WPD result of the closing-spring relaxation case. (**a**) *W*_3_^0^; (**b**) *W*_3_^1^; (**c**) *W*_3_^2^; (**d**) *W*_3_^3^; (**e**) *W*_3_^4^; (**f**) *W*_3_^5^; (**g**) *W*_3_^6^; (**h**) *W*_3_^7^.

**Figure 13:**
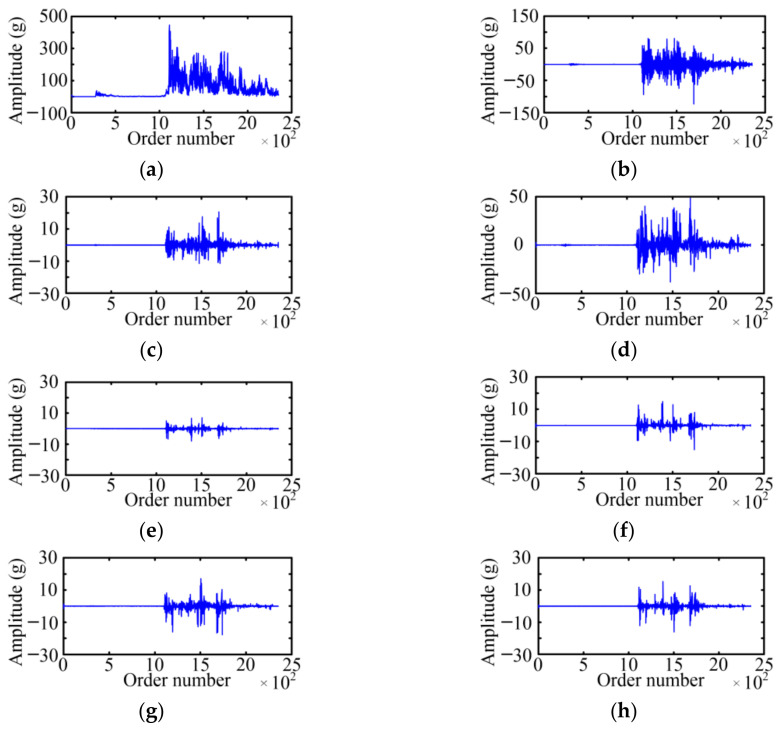
The WPD result of the reducing control voltage case. (**a**) *W*_3_^0^; (**b**) *W*_3_^1^; (**c**) *W*_3_^2^; (**d**) *W*_3_^3^; (**e**) *W*_3_^4^; (**f**) *W*_3_^5^; (**g**) *W*_3_^6^; (**h**) *W*_3_^7^.

**Figure 14:**
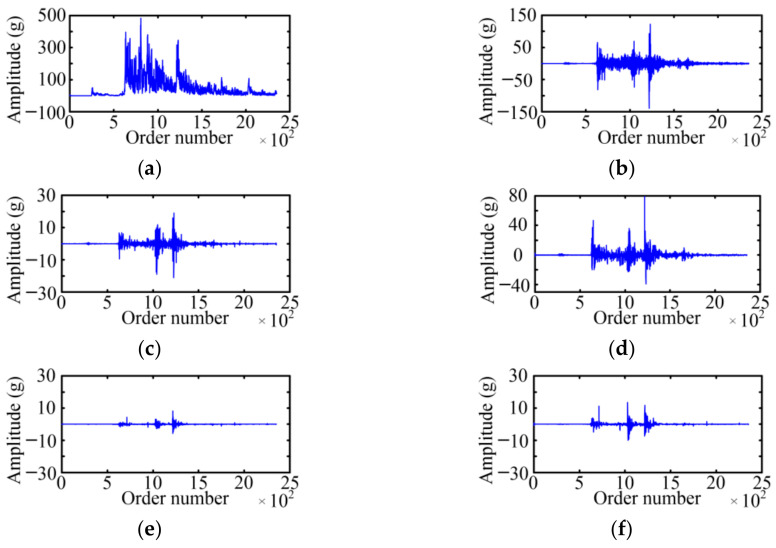

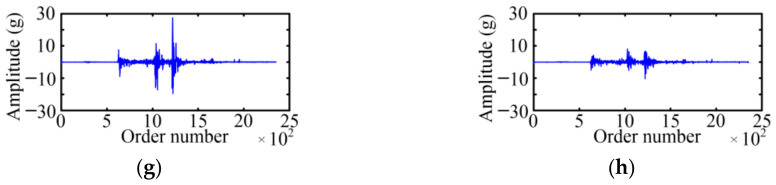
The WPD result of the relay high resistance. (**a**) *W*_3_^0^; (**b**) *W*_3_^1^; (**c**) *W*_3_^2^; (**d**) *W*_3_^3^; (**e**) *W*_3_^4^; (**f**) *W*_3_^5^; (**g**) *W*_3_^6^; (**h**) *W*_3_^7^.

Using the same method, the three-layer WPD results of the vibration signal envelop under five fault cases of the MVCB can be obtained, as shown in Figure 10, Figure 11, Figure 12, Figure 13 and Figure 14. It is easy to find that the WPD results of five cases are different in each wavelet coefficient. Compared with the normal case, each wavelet coefficient of the three fault cases is less than the corresponding wavelet coefficient of the normal state.

From (5), the energy of each frequency band after WPD under the six cases of the MVCB can be calculated, and it can be known that the energy corresponding to each frequency band changes greatly. Equation (6) is used to calculate the percentage of the energy of each frequency band in the total energy of the WPD in the six cases. Database 1 is selected for offline training of the fault feature, and the typical feature vector *M* is further obtained by (7) and (8). As shown in Table 6, *M*_1_, *M*_2_, *M*_3_, *M*_4_, *M*_5,_ and *M*_6_ are the typical feature vector of six cases, respectively.

In order to confirm if the suggested strategy is effective, Database 2 is further used as the online test data, and the Euclidean distance between the feature vector *T* of each online operational vibration signal and the typical fault feature vector *M* is calculated. In accordance with the principle of closest distance, the fault diagnosis of 30 groups of online test data can be discriminated.

**Figure 15:**
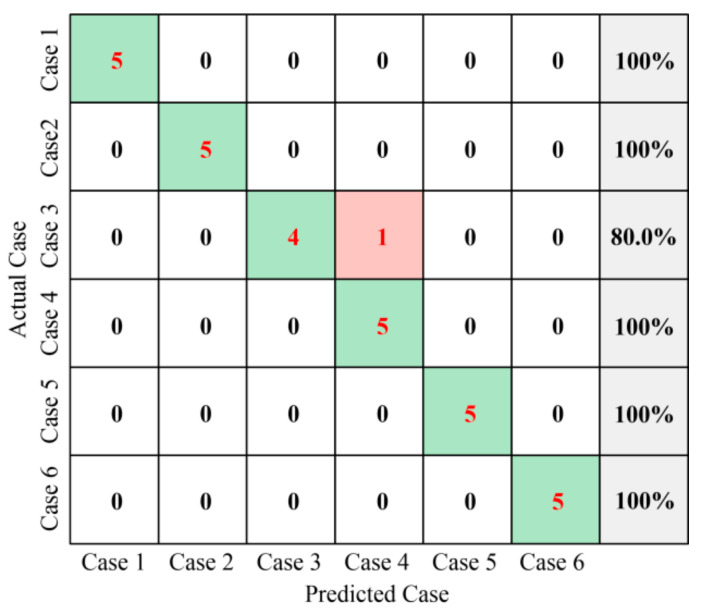
Fault diagnosis results of online test data.

The multiclass confusion matrix is used to show the difference between the actual case and the predicted case. Figure 15 shows the multiclass confusion matrix for the proposed approach. The multiclass confusion matrix provides detailed documentation of the classification outcomes for each case, including information on both correct and incorrect classification. The vertical axis and horizontal axis of the multiclass confusion matrix each represent the actual and predicted cases, respectively.

It should be noted that some pieces are different from those on the main diagonal. The element values in the last column of the multiclass confusion matrix represent the classification accuracy of each case. In the scenario depicted in Figure 15, we can see that Case 3 has the lowest precision, which is 80%. For Case 1, Case 2, Case4, Case 5 and Case 6, the fault diagnosis is 100%. Additionally, the fault diagnosis precision of the proposed approach is 96.67% for all test samples, demonstrating that high performance is achieved for the fault diagnosis of the MVCB.

Based on the desktop computer (Intel(R) Core (TM) i9-10900K CPU@3.70 GHz 24 GB RAM), the influence of WPD layers on the precision and execution time of the proposed approach is studied. As shown in Table 7, with the WPD layers increasing from 1 to 3, the precision of the proposed approach increases from 80% to 96.67%. As the amount of WPD layers increases to 4 or 5, the accuracy remains unchanged at 96.67%, but the time for fault diagnosis also increases.

The efficiency of the proposed approach is compared with two traditional typical methods. The wavelet packet energy spectrum is directly applied to extract the feature vector of the vibration signal, and two classification methods (ELM and back propagation neural network (BPNN)) are used to treat the online data. As shown in Table 8, the accuracy of the proposed approach is better than that of the other two typical methods, and the execution time of the proposed approach is less than that of the other two methods.

## 5. Conclusions

The fault diagnosis of the MVCB based on vibration signal envelop analysis is proposed in this paper. Firstly, the vibration signals of the MVCB during closing operation under four cases are collected by the data acquisition system. Secondly, the Hilbert transform is used to extract the envelope of the vibration signal, and the fault feature of the vibration signal envelope is acquired by the wavelet packet energy spectrum. Then, the fault feature library is built by the fault feature and the label of the historical vibration signal, and the typical fault feature vector *M* is calculated by the fault feature library. After that, the online collecting operational vibration signal is extracted from the envelope by the Hilbert transform, and the fault feature vector is acquired from the wavelet packet energy spectrum to gain the fault feature vector *T*. Finally, the fault state is classified by distance discrimination. The proposed fault diagnosis approach based on vibration signal envelope analysis reduces the expenditure on software and hardware which is applicable for the online fault diagnosis of the MVCB. Till now, the proposed fault method has only undergone laboratory validation. In the future, the practical deployment and application of the proposed fault diagnosis method will be our main tasks.

## Figures and Tables

**Table 1:** Configuration of data acquisition system.

Device Name	Manufacturer	Type	Configuration	
			Parameters	Value
Vibration sensor	Lance	LC0158T	Range	166 g
			Sensitivity	30.1 mV/g
Acquisition card	Shuangnuo	MP4221	Resolution	12 bit
			Sampling frequency	125 kHz
Power supply	Mornsun	LM35	Output voltage	+24 V

**Table 2:** Typical cases of MVCB.

	Case	Fault Reason	Emulation
Case 1	Normal	-	-
Case 2	Iron core jamming	Iron corrosion	Suspending heavy weight
Case 3	Bearing-screw looseness	Vibration	Loosening bearing screw
Case 4	Closing-spring relaxation	Spring aging	Adjusting spring-end nuts
Case 5	Low relay coil voltage	Low control voltage	Reducing control voltage
Case 6	Relay high resistance	Coil aging	Adding a resistor in relay coil

**Table 3:** Summarizing database of MVCB.

Case	Amount	Database 1	Database 2	Description
Case 1	15 sets	10 sets	5 sets	Normal
Case 2	15 sets	10 sets	5 sets	Iron core jamming
Case 3	15 sets	10 sets	5 sets	Bearing-screw looseness
Case 4	15 sets	10 sets	5 sets	Closing-spring relaxation
Case 5	15 sets	10 sets	5 sets	Low relay coil voltage
Case 6	15 sets	10 sets	5 sets	Relay high resistance

**Table 4:** Feature definition for spectrum diagram.

Order	Feature Name	Feature Express
1	Average value of the frequency spectrum	$C_{avf}=\frac{1}{L}\sum_{f=1}^{L}S(f)$
2	Root mean square value of the frequency spectrum	$C_{rmsf}={\left(\frac{1}{L}\sum_{f=1}^{L}S^{2}(f)\right)}^{1/2}$
3	Standard deviation value of the frequency spectrum	$C_{sdf}={\left(\frac{1}{L}\sum_{f=1}^{L}{\left(S(f)-C_{avf}\right)}^{2}\right)}^{1/2}$
4	Center of gravity value of the frequency spectrum	$C_{gcf}=(\sum_{f=1}^{L}f\times S(f))/\sum_{f=1}^{L}S(f)$

**Table 5:** Fault diagnosis based on frequency domain features.

	Case 1	Case 2	Case 3	Case 4	Case 5	Case 6	Average
Training accuracy (%)	90%	90%	100%	100%	100%	70%	91.67%
Testing accuracy (%)	80%	60%	100%	80%	60%	40%	76.67%

**Table 6:** Typical feature vector of six cases.

	*m*_1_	*m*_2_	*m*_3_	*m*_4_	*m*_5_	*m*_6_	*m*_7_	*m*_8_
*M*_1_	0.7635	0.1180	0.0202	0.0544	0.0053	0.001	0.0163	0.0124
*M*_2_	0.7880	0.0915	0.0166	0.0509	0.0130	0.0143	0.0137	0.0121
*M*_3_	0.8193	0.0897	0.0176	0.0427	0.0040	0.0052	0.0148	0.0066
*M*_4_	0.8132	0.0979	0.0140	0.0425	0.0043	0.0086	0.0112	0.0084
*M*_5_	0.7463	0.1263	0.0198	0.0563	0.0078	0.0138	0.0158	0.0139
*M*_6_	0.7606	0.1198	0.0195	0.0517	0.0068	0.0127	0.0164	0.0125

**Table 7:** Impact of decomposition layers on accuracy and execution time.

	Configuration				
Computer	Windows 10, CPU i9@3.70 GHz, 24 GB RAM				
Tool	MATLAB 2020b				
Decomposition layers	1	2	3	4	5
Accuracy (%)	80%	86.67%	96.67%	96.67%	96.67%
Time (s)	0.0089	0.0095	0.0131	0.0162	0.0191

**Table 8:** Comparisons of different methods.

Method	Case 1	Case 2	Case 3	Case 4	Case 5	Case 6	Accuracy (%)	Time (s)
Proposed method	100%	100%	80%	100%	100%	100%	96.67%	0.0131
WPD + ELM	40%	100%	100%	100%	80%	100%	86.67%	0.0291
WPD + BPNN	60%	100%	100%	100%	100%	80%	90%	0.0323

## Data Availability

The data supporting the findings of this study are available from the first author or corresponding author upon reasonable request.
